# Incidence and outcomes of patients admitted to emergency departments with urinary tract infections in Denmark: a retrospective cohort study

**DOI:** 10.1080/07853890.2025.2546059

**Published:** 2025-08-19

**Authors:** Ida Christine Schütt, Gustav Emil Hansen, Alexander Koch-Pedersen, Annmarie Lassen, Flemming Schønning Rosenvinge, Christian Backer Mogensen, Helene Skjøt-Arkil, Lone Wulff Madsen, Isik Somuncu Johansen

**Affiliations:** ^a^ Department of Infectious Diseases, Odense University Hospital, Odense, Denmark; ^b^ Department of Emergency Medicine, Odense University Hospital, Odense, Denmark; ^c^ Department of Clinical Research, University of Southern Denmark, Odense, Denmark; ^d^ Department of Clinical Microbiology, Odense University Hospital, Odense, Denmark; ^e^ Department of Emergency Medicine, University Hospital of Southern Denmark, Aabenraa, Denmark; ^f^ Department of Regional Health Research, University of Southern Denmark, Odense, Denmark; ^g^ Lille Baelt Hospital, Department of Medicine, Kolding, Denmark; ^h^ Research Unit of Infectious Diseases, Department of Clinical Research, University of Southern Denmark, Odense, Denmark

**Keywords:** Admission, incidence, outcome, UTI, urinary tract infection

## Abstract

**Background:**

Urinary tract infection (UTI) is a common Emergency Department (ED) diagnosis and cause of hospitalization. This study aimed to assess the incidence of UTI-related referrals and admissions in the Region of Southern Denmark and describe patient demographics, clinical and laboratory findings, readmission rates and mortality.

**Materials and methods:**

This retrospective cohort study included all referrals to five EDs in the Region of Southern Denmark from January 1, 2016, to March 19, 2018. Patients aged ≥18 years, with a UTI discharge diagnosis were included.

**Results:**

A total of 3,754 individual UTI patients were identified, corresponding to an incidence of 17.6 per 10,000 person-years and an admission rate of 10.8 per 10,000 person-years. Admitted patients were older (median age: 77 years), had more comorbidities, higher CRP and leukocyte levels (*p* < .001). *E. coli* was the most frequent pathogen in both urine (66.6%) and blood cultures (53.6%). Admission was associated with male sex (OR 1.32), age 60**–**80 years (OR 3.07), ≥81 (OR 3.60), Charlson Comorbidity Index (CCI) ≥3 (OR 1.34), qSOFA = 1 (OR 1.56), and initiation of antibiotic treatment (OR 4.08). Thirty-day mortality was 7.4%, with age ≥81 (OR 3.86), CCI ≥3 (OR 2.73), and qSOFA = 3 (OR 5.49) as significant risk factors. Among patients who were not admitted at the initial contact, 5.1% required hospitalization within 30 days.

**Conclusions:**

UTIs are a common cause of hospitalization and represent a substantial healthcare burden in Denmark. Older age, male sex, comorbidities, and severe clinical presentations were key predictors of admission and mortality.

## Key Messages

Urinary tract infections (UTIs) represent one of the most common infectious diseases prompting emergency department visits and antibiotic prescriptions.

Older age, male sex, comorbidities, and severe clinical presentations are key predictors of admission and mortality in patients admitted to emergency departments with UTI in Denmark.

## Introduction

Urinary tract infections (UTIs) are the second most common infectious diagnosis among patients referred to the emergency departments (EDs) in Denmark [1,2]. According to the Global Burden of Diseases (GBD), there were an estimated 400 million cases of UTIs globally in 2019, with a 60.4% rise in incidence and a 140.2% increase in the number of deaths compared to 1990 [3]. In the United States, the National Center for Health Statistics reported around 10.5 million outpatient visits annually for UTI-related symptoms, with 21.3% of these visits occurring in emergency departments [4]. Similarly, a nationwide study in Sweden revealed that urogenital infections account for 17% of all hospitalizations due to infectious diseases, with a steady rise in cases observed between 1998 and 2019 [5]. The increasing burden of UTI-related healthcare contacts has been attributed to an aging population and an increasing prevalence of predisposing conditions [6,7].

The rise in antibiotic resistance has been closely linked to treatment of UTIs both worldwide and in Denmark [8–10]. In Denmark, the consumption of penicillins combined with beta-lactamase inhibitors has increased by 89.5%, since 2014 [10]. Concurrently, resistance to piperacillin/tazobactam in invasive infections caused by *Escherichia coli* and *Klebsiella pneumoniae* has risen, reaching 10% in 2023 [10]. Despite national and regional variations in recommended antibiotic regimens, it is crucial that physicians adhere to established antibiotic guidelines to minimize the unnecessary use of broad-spectrum antibiotics. A Danish study has reported guideline-concordance as low as 37% for hospitalized UTI cases [11]. Similar patterns of non-adherence have been observed internationally, leading to an increased reliance on broad-spectrum empirical therapy, which may drive antimicrobial resistance without improving clinical outcomes [12,13].

A comprehensive understanding of UTI hospitalization trends is essential for optimizing healthcare resource allocation, improving diagnostic accuracy and guiding treatment decisions in the ED. Despite the high prevalence of UTIs, population-based data on the incidence, patient characteristics, and clinical outcomes of UTIs requiring hospital referral remain limited. Therefore, this study aimed to estimate the incidence rates of acute referrals and hospital admissions due to UTIs among adults in the Region of Southern Denmark. The secondary objectives were to: i) describe the demographic, clinical-, laboratory characteristics and the empiric antibiotic treatment, of admitted versus non-admitted patients; ii) estimate 30-day mortality rates; and iii) determine the admission rate among patients not initially admitted.

## Materials and methods

### Study design and setting

We conducted a population-based, multi-center, retrospective cohort study of all acute adult hospital contacts in the Region of Southern Denmark. The study included patients evaluated at four 24-hour EDs which receive patients either by ambulance or through referrals from general practitioners. A previous study have presented basic and prognostic data of the cohort [14]. Healthcare in Denmark is free and accessible to all citizens.

The study was reported in accordance with the STROBE (The Strengthening the Reporting of Observational Studies in Epidemiology) [15].

### Population

All residents in Denmark have a unique personal identification number (PIN) that enables the linkage of individual data across multiple national healthcare registers. We included all adult patients (≥18 years) with a valid PIN who were registered with a primary UTI discharge diagnosis at an ED in the Region of Southern Denmark between January 1, 2016, and March 19, 2018, covering a population of 968,863 adults. The International Classification of Diseases, 10th revision (ICD-10) codes for UTI are shown in Supplementary Table S1. For patients with multiple UTI contacts within the study period, only the incident contact was used for analysis.

### Data sources

Information regarding time and date for all ED visit, prescribed antibiotics, results of urinary dip stick and measured vital values was drawn from the electronic patient administrative system. Information was linked to the Danish Civil Registration System and the National Patient Registry (NPR) resulting in enrichment of data with death, discharge diagnosis, treatment procedures, comorbidities, readmission and length of hospitalization. Microbiological results from initial blood and urine samples (within 48 h from the time of referral) were obtained from the local Microbiology Database [16,17]. Blood test results were extracted from the regional laboratory databases.

### Definitions

Hospital admission was defined as a hospital stay lasting more than 24 h. The Charlson Comorbidity Index (CCI) was calculated based on discharge diagnoses (ICD-10 codes) recorded in the NPR during the 10 years prior to the UTI diagnosis as described by Quan, Li et al. [18].

The qSOFA score was calculated using measurements of respiratory rate, systolic blood pressure, and Glasgow Coma Scale score recorded by nursing staff upon the patient’s arrival at the ED [19]. Participants with one or more missing qSOFA parameters were classified as having a missing qSOFA score and are presented as a separate category (Tables 1 and 2).

**Table 1.** Baseline demographic and clinical characteristics of patients with urinary tract infections.

	Non-admitted *n* = 1 453	Admitted *n* = 2 301	*P* value
Median age	68	77	<0.001^b^
Sex, *n* (%)			<0.001^a^
Female	942 (64.8)	1140 (49.5)	–
Male	511 (35.2)	1161 (50.5)	–
Comorbid conditions, *n* (%)			
Cerebrovascular disease	232 (15.97)	479 (20.82)	<0.001^c^
Any malignancy, incl. leukemia and lymphoma	201 (13.83)	411 (17.86)	0.001^c^
DM without complications	166 (11.42)	409 (17.77)	<0.001^c^
Chronic obstructive pulmonary disease	157 (10.81)	316 (13.73)	0.009^c^
Congestive heart failure	82 (5.64)	235 (10.21)	<0.001^c^
Peripheral vascular disease	81 (5.57)	222 (9.65)	<0.001^c^
Dementia	107 (7.36)	185 (8.04)	0.466^c^
Moderate-severe renal disease	62 (4.27)	200 (8.69)	<0.001^c^
Acute myocardial infarction	66 (4.54)	166 (7.21)	0.001^c^
DM with complications	60 (4.13)	167 (7.26)	<0.001^c^
Peptic ulcer disease	59 (4.06)	105 (4.56)	0.474^c^
Connective tissue disease	45 (3.10)	119 (5.17)	0.003^c^
Mild liver disease	30 (2.06)	60 (2.61)	0.295^c^
Metastatic solid tumor	20 (1.38)	58 (2.52)	0.017^c^
Hemiplegia	25 (1.72)	45 (1.96)	0.612^c^
Moderate-severe liver disease	8 (0.55)	22 (0.96)	0.176^c^
HIV or AIDS	<5	<5	0.427^c^
Charlson comorbidity index, *n* (%)			<0.001^a^
0	900 (61.9)	1 130 (49.1)	
1–2	384 (26.4)	751 (32.6)	
>2	169 (11.6)	420 (18.3)	
Vital parameters, mean (SD), *n*			
Temperature	37.36 (0.98), 611	37.88 (1.16), 1385	<0.001^d^
Systolic blood pressure	136.02 (22.31), 717	130.37 (23.70), 1619	<0.001^d^
Respiratory rate	17.86 (4.50), 676	19.52 (5.06), 1556	<0.001^d^
Glasgow coma scale	14.79 (0.89), 569	14.63 (1.11), 1399	0.003^d^
qSOFA score, *n* (%)			<0.001^a^
0	395 (27.2)	757 (32.9)	
1	112 (7.7)	446 (19.4)	
2	>20 (<2.0)	114 (5.0)	
3	<5 (<0.4)	22 (1.0)	
Missing	921 (63.4)	962 (41.8)	

AIDS acquired immunodeficiency syndrome; HIV human immunodeficiency virus; qSOFA quick sepsis related organ failure assessment; SD standard deviation.

^a^Fischer’s exact test.

^b^Median variable test.

^c^Proportion test.

^d^Mean *t* test.

**Table 2.** Odds ratios for hospital admission and 30-days all-cause mortality from multiple logistic regression.

Covariate	OR of admission (95% CI)	*p* value	OR of death within 30 days (95% CI)	*p* value
Sex				
Female	Ref		Ref	
Male	1.32 (1.13–1.55)	<0.001	1.16 (0.86–1.56)	0.33
Age (years)				
18–38	Ref		–	–
39–59	1.88 (1.42–2.49)	<0.001	**–**	–
60–80	3.07 (2.40–3.93)	<0.001	Ref: 18–80	
81+	3.60 (2.79–4.65)	<0.001	3.86 (2.81–5.30)	<0.001
Charlson comorbidity index (CCI)				
0	Ref		Ref	
1–2	1.20 (1.01–1.42)	0.04	2.13 (1.49–3.03)	<0.001
3+	1.34 (1.07–1.68)	0.01	2.73 (1.85–4.02)	<0.001
qSOFA score				
0	Ref		Ref	
1	1.56 (1.20–2.01)	<0.001	2.28 (1.45–3.57)	<0.001
2	1.65 (1.01–2.70)	0.05	3.49 (1.94–6.26)	<0.001
3	2.03 (0.58–7.05)	0.27	5.49 (1.97–15.26)	<0.01
Missing	0.93 (0.78–1.10)	0.40	1.87 (1.23–2.83)	<0.01
Administered antibiotics, *n*				
No antibiotics	Ref		Ref	
Antibiotics	4.08 (3.48–4.79)	<0.001	1.54 (1.10–2.15)	0.33

OR odds ratio; CI confidence interval; ref reference.

A positive UTI dipstick was defined as ≥1 + leukocytes. A positive urine culture was defined as ≥10^4^ bacteria/mL of urine, and a positive blood culture sample was defined as the growth of bacteria in one or more blood culture bottles.

Bacteria were grouped according to Gram stain and morphology and bacterial findings below five could not be quantified.

Antibiotics were classified according to Anatomical Therapeutic Chemical (ATC) classification (Table S2).

### Statistical methods

The incidence rates of patients admitted with UTIs per 10,000 person-years (PY) at risk were calculated using the total number of persons aged ≥18 years living in the Region of Southern Denmark [1]. The population size was estimated based on the numbers on January 1, 2016, and the follow-up time was initiated on January 1, 2016 and ended on March 19, 2018.

We summarized continuous variables as means (standard deviation), or medians with interquartile ranges (IQR) if the variables deviated from a normal distribution. Categorical variables were presented as frequencies and percentages. Statistical significance was assessed using Fishers exact test, median test, Chi-squared test, or *t* test for means whenever applicable.

Logistic regression analyses were performed to identify potential risk factors for hospital admission and 30 days mortality among UTI patients. Results are reported as odds ratios (OR) with 95% confidence intervals (CI).

A *p* value of <0.05 was considered to be statistically significant. STATA Version 17 was used for all analyzes [20].

### Ethics

The permission to collect and use the data for scientific purpose was given by the Danish Patient Safety Authority (no. 3-3013-2272/1), and the processing of personal data was notified to and approved by the Region of Southern Denmark and listed in the internal record (17/24904, 20/24502) cf. art 30 of The EU General Data Protection Regulation. The study is conducted according to the Helsinki Declaration. Ethical approval is not required for registry-based studies in Denmark

## Results

### Incidence rates

**Figure 1.**
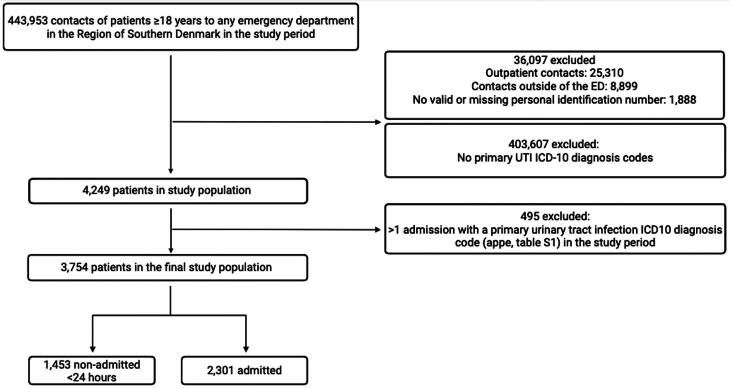
Flowchart of patient population with primary diagnoses of UTI – produced with biorender.com.

During the study period, 443,953 acute contacts were recorded (Figure 1). Of these, 36,097 (8.1%) were excluded for the following reasons: outpatient contacts (*n* = 25,310), acute contacts outside of the ED (*n* = 8,899), erroneous or missing PINs (*n* = 1,888). Among the remaining 407,856 contacts, 4,249 (1.0%) had a relevant ICD-10 code for UTI as the primary diagnosis. After excluding 495 (1.2%) to ensure only incident contacts within the period, 3,754 unique patients were included in the final analysis, yielding an overall incidence rate of 17.6 per 10,000 PY.

**Figure 2.**
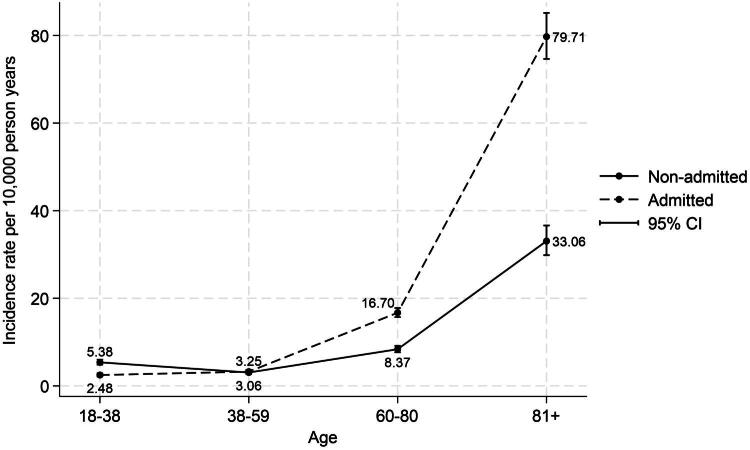
Incidence rate for all patients (men and women) stratified by age and hospital admission.

A total of 2,301 (61.3%) patients were admitted to the hospital, corresponding to incidence rates of 11.0 and 10.6 per 10,000 PY for males and females, respectively. Incidence rates stratified by age and hospital admission are reported in Figure 2. A steep increase in UTI incidence for both admitted and non-admitted with increasing age was demonstrated.

### Admitted versus non-admitted patients

Patient characteristics are shown in Table 1. Admitted patients had a higher median age, a greater proportion of men, and generally more comorbidities compared to non-admitted patients. Additionally, upon referral, admitted patients exhibited a higher mean temperature, and significantly more abnormal vital parameters and higher qSOFA scores.

The laboratory characteristics, treatment, and mortality outcomes stratified by admission status are shown in Table 3. Admitted patients had significantly elevated CRP, leukocytes, lactate and creatinine levels. Of those hospitalized, 82.2% had a urine culture and 77.4% a blood culture performed within 48 h. In urine cultures, *E. coli* was the dominant pathogen (53.4% in admitted vs 62.5% in the non-admitted, *p* < 0.001). Positive blood cultures were twice as frequent in admitted patients as in non-admitted patients (23.6% vs. 10.3%), with *E. coli* being the most frequently isolated pathogen in blood (56%). Among non-admitted patients with bacteremia, 48% were subsequently admitted within 10 days. Additionally, the same bacterial species was identified in the corresponding urine culture in 28.6% of bacteremic patients.

**Table 3.** Laboratory characteristics, mortality, and length of stay for patients with urinary tract infections.

	Non-admitted *n* = 1 453	Admitted *n* = 2 301	*p* value
Blood test, median (IQR), *n*			
C-reactive protein	23 (5.4–70), 1391	94 (32–184), 2297	<0.001^b^
Lactate	1.1 (0.7–1.5), 199	1.2 (0.8–1.8), 942	0.036^b^
Creatinine	75 (63–93), 1389	95 (72–131), 2297	<0.001^b^
Leukocytes	9.63 (7.29–12.5), 1390	12 (9.28–16.2), 2297	<0.001^b^
Urine dipstick, *n* (%)	632 (43.5)	1456 (63.3)	
Positive	523 (82.8)	1214 (83.4)	<0.001^c^
Urine culture, *n* (%)	1076 (74.1)	1889 (82.1)	
Positive	524 (48.7)	986 (52.2)	<0.001^a^
Pathogen in urine culture, *n* (%)			
*Escherichia coli*	328 (62.5)	527 (53.4)	<0.001^c^
*Klebsiella pneumoniae*	40 (7.6)	85 (8.6)	0.51^c^
*Enterococcus faecalis*	22 (4.2)	66 (6.7)	0.049^c^
*Pseudomonas aeruginosa*	7 (1.3)	54 (5.5)	<0.001^c^
Blood culture, *n* (%)	631 (43.4)	1780 (77.4)	
Positive	65 (10.3)	420 (23.6)	<0.001^a^
Pathogen in blood culture, *n* (%)			
*Escherichia coli*	26 (40)	234 (55.7)	0.018^c^
*sCoagulase negative staphylococcus*	6 (9)	22 (5)	0.72^c^
*Klebsiella pneumoniae*	<5	37 (8.8)	0.47^c^
*Staphylococcus aureus*	<5	19 (4.5)	0.59^c^
*Enterococcus faecalis*	<5	13 (3.1)	0.49^c^
*Pseudomonas aeruginosa*	<5	9 (2.1)	0.23^c^
Administered antibiotics, *n* (%)^y^	451 (31.0)	1561 (67.8)	
Penicillin with extended spectrum	303 (67.2)	565 (36.2)	
Piperacillin/tazobactam	65 (14.4)	613 (39.3)	
Cephalosporins	18 (3.9)	115 (7.4)	
Aminoglycosides	6 (1.3)	89 (5.7)	
Quinolones	28 (6.2)	58 (3.7)	
Trimethoprim and sulfamethizole	16 (3.5)	25 (1.6)	
Carbapenems	<5	16 (1.0)	
Beta-lactamase resistant penicillins	<5	7 (0.4)	
Others	8 (1.8)	35 (2.2)	
Mortality and length of stay, *n* (%)			
Length of hospital stay, median (IQR)	0 (0–0)	4 (2–7)	<0.001^d^
30 days mortality	34 (2.3)	170 (7.4)	<0.001^,c^

IQR interquartile range.

^a^Fischers exact test. ^b^Median variable test.

^c^Proportion test.

^d^Mean *t* test.

y List of other antibiotics and relevant ATC codes are found in Table S2.

The choice of antibiotics varied significantly between the two groups. Non-admitted patients predominantly received penicillin with extended spectrum (67.2% vs. 36.2%), while admitted patients had higher use of piperacillin/tazobactam (39.3% vs. 14.4%), cephalosporins (7.4% vs 3.9%), and aminoglycosides (5.7% vs. 1.3%) (Table 3).

The median length of hospital stay for admitted patients was 4 days (IQR 2-7). Overall, 30-day mortality rates were significantly higher among admitted patients (7.4% vs. 2.3%, *p* < .001). Among non-admitted patients, the rates of readmission with UTI were 3.4% within 7 days and 5.1% within 30 days.

### Factors associated with admission and mortality

In the adjusted logistic regression analysis, male sex (OR 1.32, 95% CI 1.13–1.55), age 60–80 years (OR 3.07, 95% CI 2.40–3.93), age ≥81 years (OR 3.60, 95% 2.79–4.65) and antibiotic administration (OR 4.08, 95% CI 3.48–4.79) were significantly associated with patient admission. Risk factors for 30-day mortality included age ≥ 81 (OR 3.86, 95% CI 2.81–5.30), a CCI≥ 3 (OR 2.73, 95% CI 1.85–4.02), and a qSOFA = 3 (OR 5.49, 95% CI 1.97–15.26) (Table 2).

## Discussion

In this population-based, multi-center, retrospective cohort study, we observed an overall incidence rate of 17.6 per 10,000 PY for UTI-related adult referrals to EDs in the Region of Southern Denmark. The incidence rate of admission was 10.7 per 10,000 PY, with similar rates between sexes but increasing with age. Admitted patients were older, more likely to be male, and had higher comorbidity score as well as more frequent qSOFA score ≥1 at baseline, indicating greater illness severity. Notably, the diagnostic workup at baseline was suboptimal and antibiotic selection varied considerably. The 30-day mortality rate among admitted patients strongly associated with advanced age, higher comorbidity index and elevated or missing qSOFA scores. Among non-admitted patients, 5.1% required subsequent admission within 30 days.

Globally, comparable studies have reported varying incidence rates of UTI-related hospitalizations, with rates of 18.4 per 10,000 PY in the USA and 6.8 per 10,000 PY for men and 12.4 per 10,000 for women in Japan [7,21] Incidence rates, patient demographics, and outcomes across studies are likely attributable to differences in healthcare systems, patient populations and diagnostic criteria for UTIs used in different countries [6,7,21]. Several methodological factors in our study may have contributed to an underestimation of incidence rates. Specifically, to ensure a more homogeneous cohort, repeated ED presentations for UTIs beyond the first episode during the study period were excluded. Furthermore, our analysis was limited to primary diagnosis codes assigned at the time of admission, which may have excluded cases where UTIs were identified later in the clinical course or listed as secondary diagnosis.

Despite differences in patient populations, comparable studies have reported similar results, strengthening the validity of our study. Our study showed that admitted patients had a median age of 77 years, with 18.3% having a CCI >2. This finding is consistent with other studies that have reported mean ages between 72 and 74 years and 9% having a CCI >2 [6,7,21]. In-hospital mortality among UTI patients has been reported between 2% and 5% [6,7,21], whereas we observed a mortality rate of 7.4% in admitted patients.

Our findings demonstrated a significant difference in the clinical presentation between admitted and non-admitted patients. Notably, a qSOFA score ≥2 was more than three times as prevalent among admitted patients compared to non-admitted patients. Moreover, the 30-day mortality rate was significantly higher among admitted patients and associated with advanced age, higher CCI and a qSOFA ≥1 or missing score. While limited research has specifically validated the use of qSOFA scores in patients with UTIs, several studies have established its validity as a predictor of mortality and length of stay in other patient populations [19,22]. These findings indicates the potential utility of qSOFA in risk stratification for UTI patients. However, further research is needed to explore the relationship between qSOFA scores and clinical outcomes in this specific population.

Only 5.7% of admitted patients received gentamicin, while 39.3% were treated with piperacillin/tazobactam. During the study period, guidelines recommended mecillinam and gentamicin as empirical treatment for complicated UTIs without sepsis. For patients intolerant to penicillin, second generation cephalosporins or oral ciprofloxacin combined with gentamicin were recommended. Although our cohort included patients with urosepsis, the high use of piperacillin/tazobactam could not be fully attributed to sepsis cases alone, as 14.4% of non-admitted patients were also prescribed this regimen. Guidelines for urosepsis recommended either piperacillin/tazobactam or intravenous ampicillin with gentamicin. The substantial use of piperacillin/tazobactam among non-admitted patients may reflect clinician preference for its convenience in home-based intravenous therapy.

Inconsistency between guideline and clinician choice of antibiotics has previously been mentioned in other studies. A Danish study reported that only 37% of patients with suspected UTIs were treated according to guidelines [11]. Similarly, another study identified inappropriate empiric use of piperacillin/tazobactam, potentially due to physicians’ unawareness of guidelines or time constraints [23]. An Australian study on gentamicin use for UTIs also reported poor adherence to treatment guidelines [24]. Notably, patients who received empiric gentamicin had significantly shorter durations of intravenous antibiotic therapy, emphasizing the potential of guideline adherence in reducing hospital stay [24]. Factors contributing to non-adherence may include concerns about gentamicin toxicity, lack of guideline awareness, failure to record patient weight, and diagnostic uncertainty particularly in patients with comorbidities such as renal impairment that limit gentamicin use [23,24]. Additionally, with increasing antibiotic resistance, particularly resistance to piperacillin/tazobactam in invasive infections caused by *Escherichia coli* and *Klebsiella pneumoniae* now reaching 10% in Denmark [10], there is a need for new comparative clinical trials targeting UTI treatment. Such evidence would support more rational empiric antibiotic choices and strengthen antibiotic stewardship efforts.

Microbiological diagnostic workup is essential for antibiotic resistance surveillance, which serves as the foundation for rational and effective empirical antibiotic treatment strategies. In our study, prior to antibiotic initiation, urine cultures were obtained for 82.2%, and blood cultures for 77.4% of admitted patients. This suggests potential for diagnostic improvements to further support antibiotic stewardship. Consistent with previous studies, *E. coli* was the predominant pathogen in both blood and urine cultures, accounting for over half of the positive cultures in admitted patients, followed by *Klebsiella pneumoniae, Enterococcus faecalis*, and *Pseudomonas aeruginosa* [25–27]. Notably, *E. coli* was significantly less prevalent in urine cultures from admitted patients (62.5% vs. 53.4%). This aligns with previous research indicating that complicated UTIs exhibit more diverse and polymicrobial etiologies, whereas uncomplicated cystitis and pyelonephritis are predominantly caused by *E. coli* [28].

Among non-admitted patients who underwent blood culture testing, 10.3% had positive results, with *E. coli* responsible for 40%, *coagulase-negative staphylococcus* for 9%, and the remaining cases not quantified due to counts below five. Previous studies have shown that 4–6% of non-admitted patients seen in an ED were discharged with bacteremia [29,30]. Of these, only 1.6% represented true positive results, while the rest were due to contamination. This discrepancy raises questions about the distribution of positive blood cultures in our cohort and highlights the need for further investigation into the factors influencing bacteremia in non-admitted patients [30]. Additionally, exploring the correlation between bacteremia and early readmission could provide valuable insights into patient outcomes.

A key strength of our study is the comprehensive cohort, encompassing all acute ED contacts over a 26-month period, providing a representative sample across seasonal variations. The use of a unique PIN for Danish residents allowed precise linkage to national and validated registries, offering detailed data on medical history, comorbidities, antibiotic administration, and laboratory results [17].

However, our study has limitations. Overall, this is a large dataset where data from the medical record were extracted electronically. As such, errors may be present that we have not been able to verify through manual chart review. Furthermore, we excluded 0.47% of the study population due to incomplete PINs, potentially affecting representativeness. The inability to determine pre-ED antibiotic use introduces uncertainty regarding negative urine culture results. Furthermore prescriptions given to the patients for out of hospital treatment are not included in the analysis since only antibiotic given at the hospital are included in the data. The CCI was derived only from comorbidities recorded in hospital registries, which may have resulted in incomplete data. Additionally, grouping patients by diagnosis code alone may have caused misclassification, impacting accuracy. More than one third of patients had missing qSOFA scores, which may have led to an underestimation of the disease severity. Although missing qSOFA score were not associated with hospital admission, they were associated with an increased risk of mortality, suggesting potential bias. Finally, the dataset covers the period up to 2018, and changes in clinical practice or antimicrobial resistance patterns since then may limit the current generalizability of our findings.

In conclusion, this study provides population-based estimates of the incidence rates of first-time adult UTI-related referrals and admissions to the EDs in the Region of Southern Denmark, highlighting a significant healthcare burden. Our results indicate that both baseline microbiological diagnostic workup and adherence to guideline-recommended antibiotic treatment can be improved. Optimizing diagnostic practices and antimicrobial stewardship strategies is crucial for improving patient outcomes, minimizing unnecessary healthcare utilization, and mitigating the emergence of antimicrobial resistance.

## Supplementary Material

Supplemental Material

## Data Availability

Due to Danish rules on data availability, we are unable to make an anonymized dataset public. These rules are based on the Data Protection Act, imposed by The Danish Data Protection Agency. An English translation of the Data Protection Act can be found on the official website for The Danish Data Protection Agency (https://www.datatilsynet.dk/english/legislation/). For further information, the corresponding author can be contacted.
