# Supplementary material for: Incidence and outcomes of patients admitted to emergency departments with urinary tract infections in Denmark: a retrospective cohort study
Source: Ann Med. 2025 Aug 19;57(1):2546059. doi: 10.1080/07853890.2025.2546059 (PMC12366509; doi:10.1080/07853890.2025.2546059)
Supplement: Supplemental Material [file IANN_A_2546059_SM9228.docx]

**Supplementary material**

Table S1 - ICD-10 codes including in this search (primary diagnosis) for complicated cystit/pyelonefrit

| [DN109A](https://icdpedia.dk/icdCodes/search/DN109A) | Acute infectious interstitial nephritis |
| --- | --- |
| [DN109B](https://icdpedia.dk/icdCodes/search/DN109B) | Acute pyelitis |
| [DN109C](https://icdpedia.dk/icdCodes/search/DN109C) | Acute pyelonephritis |
| [DN110](https://icdpedia.dk/icdCodes/search/DN110) | Nonobstructive reflux-associated chronic pyelonephritis |
| [DN110A](https://icdpedia.dk/icdCodes/search/DN110A) | Pyelonephritis (chronic) associated with (vesicoureteral) reflux |
| [DN118A](https://icdpedia.dk/icdCodes/search/DN118A) | Nonobstructive chronic pyelonephritis NOS |
| [DN118B](https://icdpedia.dk/icdCodes/search/DN118B) | Pyelonephritis recidivans |
| [DN119](https://icdpedia.dk/icdCodes/search/DN119) | Chronic pyelonephritis NOS |
| [DN129](https://icdpedia.dk/icdCodes/search/DN129) | Pyelonephritis NOS |
| [DN136](https://icdpedia.dk/icdCodes/search/DN136) | Pyonephrosis |
| [DN200I](https://icdpedia.dk/icdCodes/search/DN200I) | Calculous pyelonephritis |
| [DN202I](https://icdpedia.dk/icdCodes/search/DN202I) | Calculous pyelonephritis with ureteric stone |
| [DN209A](https://icdpedia.dk/icdCodes/search/DN209A) | Pyelonephritis with renal calculus or stone |
| [DN300](https://icdpedia.dk/icdCodes/search/DN300) | Acute cystitis |
| [DN302](https://icdpedia.dk/icdCodes/search/DN302) | Other chronic cystitis |
| [DN308](https://icdpedia.dk/icdCodes/search/DN308) | Other cystitis |
| [DN308B](https://icdpedia.dk/icdCodes/search/DN308B) | Hemorrhagic cystitis |
| [DN308C](https://icdpedia.dk/icdCodes/search/DN308C) | Cystitis recidivans |
| [DN308E](https://icdpedia.dk/icdCodes/search/DN308E) | Cystitis cystica |
| [DN309](https://icdpedia.dk/icdCodes/search/DN309) | Cystitis, unspecified |
| [DN341](https://icdpedia.dk/icdCodes/search/DN341) | Nonspecific urethritis |
| [DN342F](https://icdpedia.dk/icdCodes/search/DN342F) | Urethritis recidivans |
| [DN390](https://icdpedia.dk/icdCodes/search/DN390) | Urinary tract infection, site not specified |
| [DN390B](https://icdpedia.dk/icdCodes/search/DN390B) | Pyuria |
| [DA419B](https://icdpedia.dk/icdCodes/search/DA419B) | Urosepsis |

Table S2 – List of antibiotics administered

| Administered antibiotics |  | ATC code | |
| --- | --- | --- | --- |
| Aminoglycosides (gentamicin) | | | J01GB03 |
| Carbapenems (meropenem) | | | J01DH02 |
| Cephalosporins (cefuroxim, cefotaxim, ceftriaxon) | | | J01DC02, J01DD01, J01DD04 |
| Beta-lactamase- resistant penicillins (dicloxacillin, flucloxacillin) | | | J01CF01, J01CF05 |
| Penicillin with extended spectrum (ampicillin, pivampicillin, amoxicillin, pivmecillinam, mecillinam) | | | J01CA01, J01CA02, J01CA04, J01CA08, J01CA11 |
| Quinolones (ciprofloxacin, moxifloxacin) | | | J01MA02, J01MA14 |
| Trimethoprim and sulfamethizole | | | J01EA01, J01EB02 |
| Piperacillin/tazobactam | | | J01CR05 |
| Others (vancomycin, metronidazol, nitrofurantoin, methanamin) | | | J01XX05, P01AB01, J01FA06, J01FA09, J01FA10 |
